# Laparoscopic cholecystectomy for melanoma metastatic to the gallbladder: is it an adequate surgical procedure? Report of a case and review of the literature

**DOI:** 10.1186/1477-7819-5-141

**Published:** 2007-12-11

**Authors:** Ugo Marone, Corrado Caracò, Simona Losito, Antonio Daponte, Maria Grazia Chiofalo, Stefano Mori, Rocco Cerra, Luciano Pezzullo, Nicola Mozzillo

**Affiliations:** 1 Department of Surgical Oncology "B", National Cancer Institute of Naples, Italy; 2 Department of Pathology, National Cancer Institute of Naples, Italy; 3 Department of Oncology "A", National Cancer Institute of Naples, Italy

## Abstract

**Background:**

Only 2% to 4% of patients with melanoma will be diagnosed with gastrointestinal metastasis during the course of their disease. The most common sites of gastrointestinal metastases from melanoma include the small bowel (35%–67%), colon (9%–15%) and stomach (5%–7%), with a median survival of 6–10 months after surgery, and 18% survival at five years. Metastatic melanoma to the gallbladder is extremely rare and it is associated with a very poor prognosis.

**Case presentation:**

We report a case of a 54-year old man presented to observation with diagnosis of 6.1 mm thick, Clark's level IV, ulcerated melanoma of the trunk, developing in the course of the disease metastatic involvement of the gallbladder as first site of recurrence, treated by laparoscopic cholecystectomy. To date only few cases of patients with metastatic melanoma of the gallbladder treated by this surgical procedure have been reported in literature.

**Conclusion:**

Gallbladder metastasis represents a rare event as a first site of recurrence. It must be considered a possible expression of systemic disease also despite radiological absence of other metastatic lesions. Laparoscopic approach has a possible therapeutic role, but open surgery has also a concomitant diagnostic purpose because gives the possibility of manual exploration of abdominal cavity, useful particularly to reveal bowel metastatic lesions, not easily identifiable by preoperative imaging examinations.

## Background

Only 2% to 4% of patients with melanoma will be diagnosed with gastrointestinal (GI) metastasis during the course of their disease [1]. The most common sites of GI metastases from melanoma include the small bowel (35%–67%), colon (9%–15%) and stomach (5%–7%), with a median survival of 6–10 months after surgery, and 18% survival at five years [2].

Metastatic melanoma to the gallbladder is extremely rare and it is associated with a very poor prognosis [3]. We report a case of patient with melanoma of the trunk developing in the course of the disease metastatic involvement of the gallbladder as first site of recurrence, treated by laparoscopic cholecystectomy. To date only few cases of patients with metastatic melanoma of the gallbladder treated by this surgical procedure have been reported in literature [4-8].

## Case presentation

**Figure 1:**
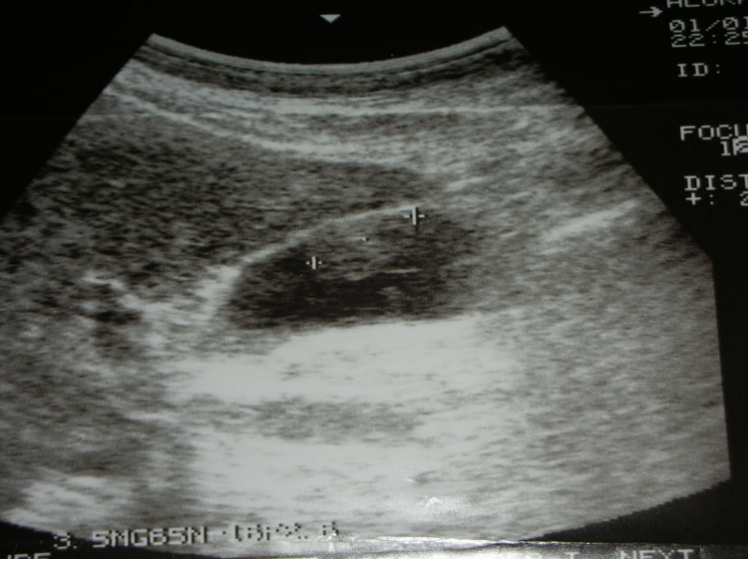
Ultrasound evidence of metastatic melanoma of the gallbladder.

**Figure 2:**
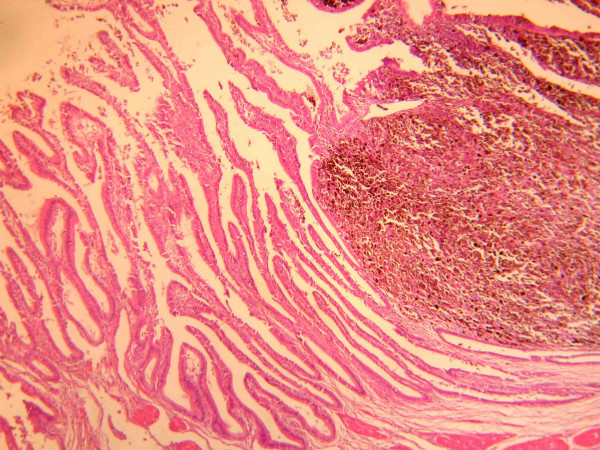
Histological appereance of gallbladder intramucous infiltration by melanoma cells stained with hematoxylin and eosin (× 100).

**Figure 3:**
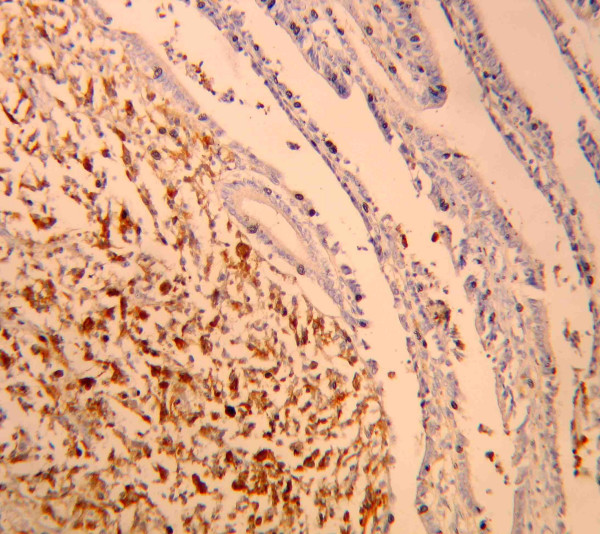
Neoplastic cells immunostained with anti-S-100 antibodies (× 400).

**Figure 4:**
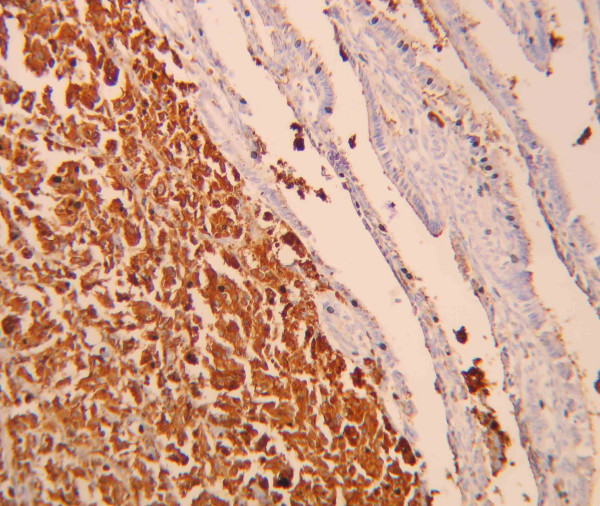
Neoplastic cells immunostained with anti-HMB-45 antibodies (× 400).

**Figure 5:**
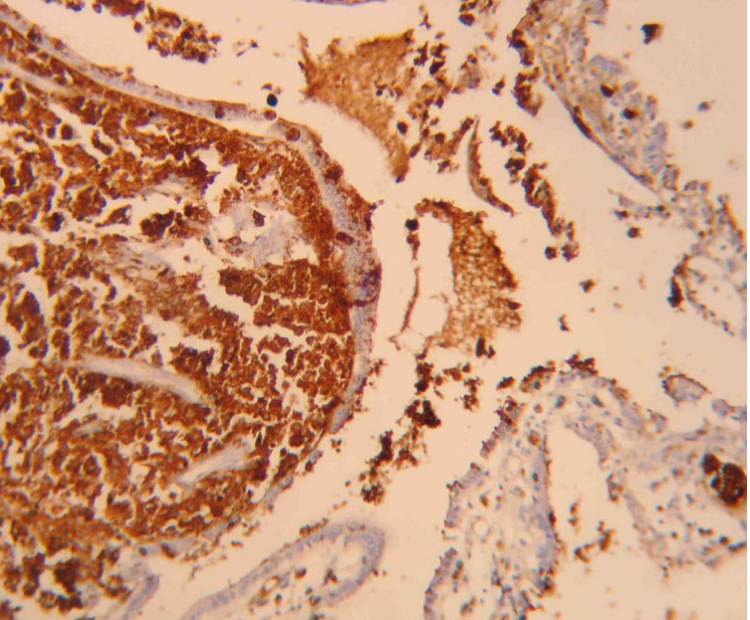
Neoplastic cells immunostained with anti-MART-1 antibodies (× 400).

In May 2001 a 54-year-old man presented to observation with diagnosis of 6.1 mm thick, Clark's level IV, ulcerated cutaneous melanoma of the trunk. Preoperative staging with chest X-ray and abdominal ultrasound (US) did not reveal signs of systemic disease. He underwent wide local excision (WEX) and sentinel lymph node biopsy (SLNB). Preoperative lymphoscintigraphy showed uptake of the radiotracer in one node of the left axilla that was removed and resulted on finally pathology negative for metastatic melanoma. The patient was submitted to regular follow-up every three months with physical examination, chest X-ray, US and blood work, according to the Multicenter Selective Lymphadenectomy Trial [9]. In January 2002 hepatobiliary US detected the presence of intracholecystic nodule of 1.0 cm. Three months later the patient presented upper abdominal pain mimicking symptomatic cholecystolithiasis. Blood analysis values were normal. US examination showed an increasing in size to 2.0 cm of the intracholecystic nodular image (Figure 1). Computerised tomography (CT) confirmed the presence of a nodule in the lumen of the gallbladder measuring about 2.0 cm and a positron emission tomography (PET) revelead uptake in the gallbladder area without evidence of systemic uptake of the radiotracer. In May 2002 the patient underwent laparoscopic cholecystectomy. Intrabdominal exploration showed a normal gallbladder but with an enlarged blue coloured lymph node along the cystic duct, and the presence of a dark spot image of the diaphragmatic peritoneum. The gallbladder was removed together with the lymph node along the cystic duct and put into an endobag to avoid any contamination of the abdominal cavity and extracted. An excisional biopsy of the diaphragmatic peritoneal lesion concluded the surgical procedure. Macroscopic evaluation of the gallbladder showed one vegetant brown coloured polyp adherent to the mucosa, projected into the lumen, measuring about 3,5 cm in size, and a brown lymph node 1.5 cm in diameter. At histological examination, the mucosal (Figure 2) and muscular layers of the gallbladder were diffusely infiltrated by epithelioid and spindle heavely pigmentated cells; the epithelium was partially eroded and no junctional activity was evident; the cystic duct lymph node and the peritoneal node were both involved by metastatic cells from melanoma. Immunohistochemical staining of the removed tissues showed a strong positivity for S 100 protein, HMB 45 and MART-1 (Figure 3, 4, 5). Postoperative course was uneventful and the patient was discharged home on postoperative day two. No adjuvant chemotherapy was offered at this time by our oncologists. Two months later the patient presented clinical evidence of intestinal obstruction. CT revealed intussusception of two segments of the small bowel tract. At laparotomy resection of two portions of the involved small bowel by two stenosing metastases with contiguous involvement of the mesentery and of mesenteric lymph nodes was performed. Pathology confirmed the abdominal recurrence by metastatic melanoma. After surgery he recieved chemoimmunotherapy (fotemustine 100 mg/m^2 ^and dacarbazine 900 mg/m^2 ^intravenously every three weeks for a total of three cycles plus interferon alfa-2b 5 MU/m^2 ^subcutaneously).

Seventeen months after SLNB, PET showed disseminated disease. The patient died shortly thereafter in April 2003, eleven months after cholecystectomy.

## Discussion

Both primary and metastatic melanoma of the gallbladder is extremely rare. The distinction between primary and metastatic lesions can be complicated on the basis of clinical, radiological and histopathological features, however the presence of a past history of melanoma in the skin or in other common primary sites combined with the absence of junctional activity (intra-epithelial extension in the mucosa overlying the tumor) orientate to the diagnosis of metastatic lesion, even if some investigators also described junctional activity in cases of metastatic involvement of the gallbladder, not considering this phenomenon sufficient to distinguish between primary and metastatic disease [10]. Moreover some authors believe that melanoma in the biliary tract is almost always a metastasis and the majority of cases described as primary are secondary to an unrecognized or regressed extrabiliary site [11-15].

Although clinically rare, melanoma metastatic to the gallbladder accounts for 30–60% of all metastatic lesions involved this site [16]. Most of patients with gallbladder metastasis are described to have widespread disease at time of diagnosis; only few patients had a first metastasis limited to the gallbladder [17]. Isolated metastatic disease to the biliary system is accompanied by obstructive jaundice if the tumor is located in the common bile duct or by upper abdominal pain mimicking symptomatic cholecystolithiasis, but asymptomatic cases were also described [4]. In contrast to gallbladder cancer, melanoma of the gallbladder does not seem associated to cholelithiasis [17].

Because of the extreme rarity of gallbladder melanoma, the diagnosis is difficult to suspect preoperatively. Nevertheless accurate investigation of unclear lesions of the gallbladder and biliary tree in patients with a past history of melanoma will be necessary [3]. Ultrasonography is the staging investigation which can revealed an intracholecystic nodule or mass preoperatively. Metastatic disease can appear as multiple flat and infiltrative lesions or as single polypoid tumor [18]. In the latter case, ultrasound appereances typical of gallbladder metastases are those of single or multiple hyperechoic masses increasing in size more than 1 cm in diameter and attached to the gallbladder wall, with minimal to absent acoustic shadowing due to their low density, and with dopplergraphic signs of blood flow [19-21]. CT scan can reveal solid masses of the biliary tract and a positivity at FDG PET can reveal metastatic unespected sites [17,7].

The prognosis of melanoma of the gallbladder is very poor. The mean survival rate for patients with primary and metastatic lesions is 20.1 months and 8.4 months respectively [10]. Because of the rarity of the primary melanomas of the gallbladder, no therapeutic guidelines can be recommended. Nevertheless, aggressive surgical therapy appears, in part, to prolong survival, and to improve the quality of life in several patients [22].

In case of metastatic lesions of the gallbladder, treatment options depend on the extension of the disease and on the clinical status of the patient. When metastases are limited to the gallbladder, surgical treatment is indicated to avoid symptoms or tumor complications and can improve prognosis significantly [5,10]. Dong *et al *reported a 100% survival at 1 year in patients who underwent surgery for isolated gallbladder metastases compared with 0% for those with unresectable tumors. In this series, among 19 patients with melanoma metastatic to the gallbladder, the median survival following presentation with disease was 7 months, with the majority of patients having multiple metastases (89%) and only 2 patients with disease confined to the gallbladder that survived more than 100 months [17]. Nevertheless, even in case of disseminated disease, surgical removal seems to be a worthwile palliative procedure [10,13].

The effectiveness of complementary chemotherapy and immunotherapy after removal of a metastasis of malignant melanoma of the gastrointestinal tract is still being examined [4]. In the case we reported, after preoperative imaging examinations and laparoscopic surgery our oncologists considered the patient free of disease, therefore no adjuvant chemotherapy was offered at this time, as usually done after surgery in absence of residual metastatic disease. After the second operation they take in consideration to administer chemoimmunotherapy as a palliative procedure for the disease progression.

Chemotherapy remains a palliative procedure for patients with stage IV melanoma and even if can improve disease free survival, does not influence 5-year overall survival rate, that in these patients is approximately 5% [23].

In the majority of cases patients with primary or secondary melanomas to the gallbladder were treated by conventional surgery. Few cases of laparoscopic cholecystectomy have been described in literature as treatment of melanoma to the gallbladder, and it is unclear when this surgical management is adequate.

Velez et al reported a case of patient with primary melanoma of the gallbladder that was removed laparoscopically, but the diagnosis was made only after histopathological examination. The patient remained disease free at last follow-up reported of 9 months after surgery [22].

Seelig et al described a case of isolated involvement of the gallbladder by metastatic melanoma. Even if visceral involvement is a sign of general metastasis, in this case there was no sign of any further metastasis, so the colecysthectomy was conducted laparoscopically with particular attention to take the gallbladder into an endobag before extraction via the periumbilical incision, in order to prevent any metastatic deposition. The patient remained disease free at last follow-up reported, 20 months after surgery [4]. Kohler *et al *reported two patients undergoing laparoscopic cholecystectomy in isolated metastasis of malignant melanoma to the gallbladder. They affirm that since the vast majority of melanoma metastases of the gallbladder are located intraluminally and lymphadenectomy in the region of the hepatoduodenal ligament does not appear to be appropriate, the operation should be carried out laparoscopically [5]. Kats *et al *reported 13 patients with a diagnosis of melanoma metastatic to the gallbladder. In this series three cholecystectomies were performed laparoscopically, with two instances of subsequent port site recurrence, even if the specimens were placed into retrieval bags prior to removal through the port sites. Five patients presented with isolated gallbladder metastases while 8 presented with disease at multiple sites. From the time of metastatic gallbladder involvement, the median survival was 12 months, with 1 patient surviving 12 years after treatment and 8 patients surviving for 1 year or more. Patients who presented with disease confined to the gallbladder had a median survival of 39 months compared to a median survival of 10 months for patients presenting with multiple metastatic sites, and among patients who underwent cholecystectomy, the median survival following treatment was 16 months, compared to 6 months for those managed not operatively. They concluded that although most patients with metastatic melanoma are not candidates for curative resection and suffer from a poor prognosis, proper selection of patients is imperative for palliative procedures. In case of metastatic gallbladder melanoma, symptomatic patients and those with melanoma confined to the gallbladder are among those who are most likely to benefit from cholecystectomy. Due to the aggressive nature and biology of advanced melanoma, minimally invasive cholecystectomy seems to be an adequate surgical option [8]. Tuveri *et al *reported a case of a patient with an isolated metastatic melanoma to the gallbladder treated by a laparoscopic cholecystectomy and lymphadenectomy of the hepatoduodenal ligament. The authors emphasize the appropiateness of a laparoscopic approach in this kind of patients, once ruled out the presence of widespread disease, with a curative intent and adequate palliation of symptoms. The patient remained free of disease at last follow-up reported of 5 years [24].

From 1996 to 2006 at the National Cancer Institute of Naples 1684 patients with diagnosis of cutaneous melanoma were surgically treated. Of these, 30 (1.7%) developed metastatic lesions to the GI tract liable to surgical exploration. Only one patient developed a gallbladder metastasis as first site of recurrence.

In the case we presented, there was no preoperatively confirmation of metastatic involvement of the gallbladder. At beginning of the operation despite a very small peritoneal nodule on the diaphragmatic cupola, there was not other evidence of disseminated disease including the normal aspect of the gallbladder. Cholecystectomy was conducted without particular difficulties, as a lythiasis operation, and the removal of the enlarged lymph node along cystic duct did not appear complicated. We utilized an endobag to remove the resected specimens. This precaution together with gentle manipulation of the gallbladder and avoidance of perforation should be practiced to help minimize the possibility of tumor seeding during laparoscopic surgery, responsible for port site metastases or peritoneal metastases [24]. No complications were recorded perioperatively and in the course of the disease that might be correlated to the laparoscopic approach adopted. The only controversial issue of the laparoscopic approach was its diagnostic purpose in absence of radiological evidence of systemic disease, because of the impossibility of a manual exploration of the bowel tract as we routinely performed at laparotomy to detect intraluminal metastatic lesions involving the mucosa, not easily identifiable preoperatively by instrumental examinations, despite of those in form of multiple serosal implants, more easily identified [23]. Anyway, metastatic lesions of the bowel may missed at manual exploration as well.

## Conclusion

Melanoma has a propensity to spread extensively, often involving multiple visceral sites. Biliary symptoms in a patient with a history of cutaneous melanoma should be investigated as possible evidence of biliary tract metastases. Gallbladder metastasis represents a rare event as first site of recurrence as occurred in the case reported in this paper. It must be considered a possible expression of systemic disease also despite radiological (CT or PET) absence of other metastatic lesions. Laparoscopic approach has a possible therapeutic role, expecially in patients with isolated metastasis of the gallbladder, but open surgery has also a concomitant diagnostic purpose because gives the possibility of manual exploration of the abdominal cavity, useful particularly to reveal bowel metastatic lesions, not easily identifiable by preoperative imaging examinations.

In absence of adjuvant therapy the surgical approach is the only treatment that may assure a better survival.

## Competing interests

The author(s) declare that they have no competing interests.

## Authors' contributions

UM conceived the study, carried out the literature search, and draft the manuscript; CC, AD and MGC helped in management of the patient and preparation of the manuscript; SL performed the histological analysis and provided histological sections as figures for the manuscript; SM, RC and LP carried out literature review and manuscript drafting; NM made critical revision and supervision.

All authors read and approved the final manuscript.
